# Secure care (forensic) hospital evaluation of manualised interpersonal art-psychotherapy (SCHEMA): A randomised controlled trial protocol

**DOI:** 10.3310/nihropenres.13801.2

**Published:** 2025-05-28

**Authors:** Simon S. Hackett, Paula Foscarini-Craggs, Katie Aafjes-van Doorn, Matthew Franklin, Muhammad Riaz, Ania Zubala, Jennifer Condie, Iain McKinnon, Arman Iranpour, Toni Leigh Harrison, Sophie Rose, Elizabeth Randell, Rachel McNamara

**Affiliations:** 1 Faculty of Medical Sciences, Baddiley-Clark Building, Richardson Road, Newcastle University Population Health Sciences Institute, Newcastle upon Tyne, England, NE2 4AX, UK; 2 Tyne and Wear NHS Foundation Trust, Jubilee Rd, Cumbria Northumberland, Newcastle upon Tyne, England, NE3 3X, UK; 3 Centre for Trials Research, Neuadd Meirionnydd, Heath Park, Cardiff University, Cardiff, Wales, CF14 4YS, UK; 4 West Yangsi Rd, New York University Shanghai, Pudong Xin Qu 567, Shanghai, 200124, China; 5 Sheffield Centre for Health and Related Research (SCHARR), School of Medicine and Population Health, Regent Court, 30 Regent Street, The University of Sheffield, Sheffield, England, S1 4DA, UK; 6 Centre for Brain Sciences, Kennedy Tower. Royal Edinburgh Hospital, Morningside Place, The University of Edinburgh, Edinburgh, EH10 5HF, UK

**Keywords:** Intellectual disability, borderline intellectual functioning, secure care, Art Psychotherapy, aggression.

## Abstract

**Background:**

Within the criminal Justice System in the UK one-third of prisoners self-identified as having a learning difficulty and/or disability. This is broadly consistent with formal assessment of the needs of offenders, with 29% of the offender population having a learning disability. In the UK, NHS and private/independent sector secure care (Forensic) provides assessment and treatment for men and women who have come into contact within the Criminal Justice System and have mental health needs, a personality disorder, and/or learning disability. Patients in these services are often detained under the Mental Health Act (1983) and/or have licence conditions that have been set by the Ministry of Justice.

Interpersonal art psychotherapy was developed within secure care as an accessible psychological intervention for adults with mild intellectual disabilities or borderline intellectual functioning. A feasibility randomised controlled trial of interpersonal art psychotherapy showed that assessment of key feasibility objectives were met and the trial procedures were acceptable, indicating progression to a definitive trial.

**Methods:**

This is a two-arm single blind randomised controlled trial of effectiveness comparing manualised interpersonal art psychotherapy and Usual Care (UC) to UC. The Randomised Controlled Trial (RCT) will be conducted in a minimum of 10 secure care hospitals (NHS & Independent) with secure care (Forensic) facilities and will recruit 150 participants. The trial design includes an integrated assessment of cost-effectiveness.

**Results:**

Individuals with intellectual disabilities and borderline intellectual functioning were involved in the design and set up of the trial. The trial is currently open to recruitment for participants from eight NHS and private/independent secure care sites in the UK.

**Conclusions:**

A full report of study results will be published on completion of the trial.

**The trial has been registered:**

ISRCTN57406593 ( ISRCTN registry, 2024). This published protocol corresponds with version 6, dated 12.08.2024.

## Plain Language Summary

**Background:**

Some people with learning difficulties (learning disabilities and borderline intellectual functioning) who commit a crime and go to court can be sent to prison or to a hospital with secure care. People with learning difficulties in secure care are more likely to stay there longer than people without a learning difficulty. Records show that people in secure care hurt either themselves or others more often than in other mental health hospitals.

Lots of psychological therapies available to help people with mental health difficulties are based on talking, which might not always be the best approach for people with learning difficulties. People who struggle with reading information and communication can find visual and art-based approaches a helpful way to understand and manage their own mental health needs.

**Intervention:**

Interpersonal art psychotherapy has been designed to help people in secure care (forensic) services with learning difficulties Within interpersonal art psychotherapy people to use creative ways to communicate and discuss this with a therapist.

**Design:**

We want to find out if interpersonal art psychotherapy is helpful and value for money for people with learning disabilities who are in secure care.

We will be testing if interpersonal art psychotherapy works better than the usual care that people receive. To do this we will need to recruit 150 people and put them into groups by chance, with half having interpersonal art psychotherapy and half will have usual care. This is called a randomised controlled trial (RCT).

**Results and Impact:**

We will find out if interpersonal art psychotherapy can help people who are in secure care to improve their mood, become less distressed, and not hurt themselves or others. We think that this research will give people with a learning difficulty more choice about accessing psychotherapy in secure care.

## Introduction

Within the Criminal Justice System (CJS) in the UK one-third of prisoners self-identified as having a learning difficulty and/or disability ( Criminal Justice Joint Inspection, 2021). This is broadly consistent with formal assessment of the needs of offenders, with 29% of the offender population having an intellectual disability/developmental disability (IDD) ( Criminal Justice Joint Inspection, 2021). The care of people with IDD within the CJS is relatively advanced in UK, however, offenders with IDD experience difficulties at all stages ( Chester, 2018).

Secure care (Forensic) provision is available in the UK NHS and private/independent sector for the assessment and treatment for men and women who have come into contact within the Criminal Justice System and have mental health needs, a personality disorder, and/or learning/intellectual disability (ID). There are three levels of security across adult secure care inpatient services, (i) high, for adults posing an immediate risk to the public and who must not be able to escape; (ii) medium, for adults presenting a serious risk of harm to others whose escape must be prevented; (iii) low, for adults who present a significant risk of harm to others whose escape must be impeded ( NHS England, 2018b).

A significant proportion of patients in secure care in England, 24% in high and 17.4% in medium, are consider ‘long stayers’, being in hospital for five or more continuous years ( Hare Duke *et al.*, 2018) with risks of experiencing adverse outcomes following discharge ( Westhead *et al.*, 2023). Patients with ID being treated on a secure care ward are more likely to stay in hospital for longer (>10 years in high secure, 5 years in medium secure or 15 years in a mix of high and medium secure settings) compared to patients on other types of secure mental health wards ( Hare Duke *et al.*, 2018).

In the UK secure care services are reported to consume a fifth of the overall mental health budget for a small share of the patient population, approximately 8000 patients, within UK mental health service ( Tully *et al.*, 2024). In a subset of this provision, secure care for people with ID, three high secure hospitals in England provide just over 700 beds and around 60 medium secure units providing around 3500 beds, with nearly 35% of those beds provided by the independent sector ( Taylor & Lindsay, 2018). The health expenditure for ID secure care is estimated at over £300 million per annum ( Morrissey *et al.*, 2017).

## Psychological interventions in secure care

There have been a range of innovations in psychosocial interventions for reducing violence and aggression ( Wigham *et al.*, 2022). Specifically in secure care, a systematic review of RCTs of psychological interventions offered to forensic/secure mental health inpatients (n=9 studies including 523 participants) reported that current practice is based on limited evidence with no consistent significant findings ( MacInnes & Masino, 2019). The study sample sizes ranged from 14 to 112. A low risk of bias assessment indicated that good quality RCTs can be undertaken within inpatient medium to high secure care settings. To our knowledge, no economic evaluations have been conducted in the reported studies ( MacInnes & Masino, 2019). Results from a systematic review and meta-analysis of controlled evaluations of psychological interventions for forensic mental health inpatients, based on 28 studies involving 1422 individuals, showed that psychological treatment had no benefit over the comparator condition on many relevant domains including impulsivity, empathy, coping skills, anger or inpatient violence ( Gilling McIntosh *et al.*, 2021). A systematic review of 23 Arts Therapies studies in forensic care, including a narrative synthesis and meta-analysis, reported significant outcomes for psychiatric symptoms and psychological and social functioning ( Abbing *et al.*, 2023). Mechanisms of change were identified as being an improvement in regulatory processes, such as self-regulation of emotion, stress regulation, impulse regulation, cognitive regulation, social regulation, behaviour regulation, and self-management. Because of their experiential, less verbal approach, arts therapies were also highlighted as being relevant for people in the criminal justice system with mild intellectual disability (MID) or borderline intellectual functioning (BIF) ( Abbing *et al.*, 2023).

## Aggressive behaviour in people with intellectual disability or borderline intellectual functioning

Aggressive behaviour and self-harm are common in people MID/BIF (IQ 50–85), particularly in secure care ( Dixon-Gordon *et al.*, 2012; Nieuwenhuis *et al.*, 2022; van Swieten *et al.*, 2024). Recommendations for complex interventions for aggressive behaviour in adults with ID include the development of effective communication and trusting relationships between service users, carers, professionals, and within staff teams as being essential to facilitate effective intervention delivery ( Royston *et al.*, 2023).

## Interpersonal art psychotherapy

Interpersonal art psychotherapy was developed within secure care as an accessible psychosocial intervention for adults with MID/BIF through utilising art-based approaches and the inclusion of relational and interpersonal components within treatment ( Hackett, 2023; Hackett & Aafjes-van Doorn, 2019; Hackett *et al.*, 2017; Hackett *et al.*, 2020). A feasibility RCT of Interpersonal Art Psychotherapy showed that assessment of key feasibility objectives were met and the trial procedures were acceptable, indicating progression to a definitive trial ( Hackett *et al.*, 2020). In addition, between-group differences of interpersonal art psychotherapy versus the delayed treatment control showed a ‘signal’ effect-size of .65 for total scores and .93 in the verbal aggression sub-scale ( Hackett *et al.*, 2020; Power *et al.*, 2023).

## Aims and objectives

The aim of this trial is to answer the following research question: Does interpersonal art psychotherapy reduce (i) the frequency and severity of aggressive incidents and/or (ii) patient self-reported distress associated with psychiatric symptoms in adults within secure care who have MID/BIF compared to usual care (UC).

### Primary objective

To assess the effectiveness of interpersonal art psychotherapy in reducing the frequency and severity aggressive behaviour in adult secure care.

### Secondary objectives

1. To determine if interpersonal art psychotherapy is cost-effective compared to UC.

2. To explore patient characteristics and psychotherapeutic processes/mechanisms within interpersonal art psychotherapy that are influential to treatment outcomes.

3. To explore the longitudinal changes in aggressive behaviour after receiving art psychotherapy.

4. To evaluate changes in patient distress relating to psychiatric symptoms

## Methods

### Patient and Public Involvement

*When and how were the patients/public first involved in the research?*

Extensive Patient and Public Involvement (PPI) consultation took place with ‘Research Abilities’ group ( Research Abilities, 2023) during the research funding application development process.

*How were the research question(s) developed and informed by their priorities, experience, and preferences?*

The testing of Art Psychotherapy as an accessible psychological treatment, less reliant upon verbal communication, was viewed as an important research topic by people with lived experience of having a learning disability or borderline intellectual functioning.

*How were patients/public involved in:*

Individual consultation meetings took place with patients in secure care. As a result, changes were made to interpersonal art psychotherapy worksheets, with greater inclusion of ‘easy read’ and visual materials.

*(a) the design and conduct of the study?*

PPI has informed the development of this research design, specifically allowing all participants who have been allocated to UC only to access the intervention after leaving the study.

*(b) choice of outcome measures?*

Measures of personal distress were included as a secondary outcome measure in response to PPI consultation with patients in secure care, this being indicated as a patient priority.

*(c) recruitment to the study?*

The Plain Language Summary was informed and revised in response to intensive engagement and detailed comments from PPI panel members at the NIHR Research Support Service (RSS). PPI consultation took place to support development of the study Participant Information Sheet (PIS), study consent form, and accessible study recruitment information video.

*How were (or will) patients/public be involved in choosing the methods and agreeing plans for dissemination of the study results to participants and linked communities?*

Additional PPI activity is planned for the development of accessible and ‘easy read’ study dissemination materials.

## Trial design

This is a two-arm single blind randomised controlled trial of effectiveness comparing manualised interpersonal art psychotherapy and UC to UC. The RCT will be conducted in a minimum of 10 secure care hospitals (NHS & Independent) with secure care (Forensic) facilities and will recruit 150 participants. The trial design includes an integrated assessment of and cost-effectiveness. Individuals with ID/BIF were involved in the design and set up of the trial.

## Participants, interventions, and outcome

### Inclusion criteria

Participants are eligible for the trial if they meet the following inclusion criteria and none of the exclusion criteria apply.

•    An inpatient in a secure hospital/unit/service with the presence of learning disability/borderline intellectual functioning indicated by either (a) meeting validated assessment criteria (recognised cognitive testing and adapting functioning assessment), or (b) a score of 57 or below on the Learning Disability Screening Questionnaire (LDSQ) ( McKenzie *et al.*, 2012) (a LDSQ copyright license has been obtained for the purposes of the study).

•    Age 18 to 60 years.

•    Able to give informed consent.

•    A current or historic HONOS (Health of the Nation Outcome Scale) ( Royal College of Psychiatrists, 2023) score between 1 and 4 for item 1 (i.e. fitting the descriptors- ‘Overactive, aggressive, disruptive, or agitated behaviour’ / ‘Behavioural problems directed at others’).

•    The participant's involvement in the study is supported by their responsible clinician and/or multidisciplinary team (MDT).

### Exclusion criteria

•    Unable to give informed consent as assessed by the clinical team.

•    Learning disability/borderline intellectual functioning not indicated based on a validated assessment or screening questionnaire (i.e. not meeting validated assessment criteria or a LDSQ Score >57).

•    A HONOS score of 0 for item 1.

•    Planned discharge within 9 months of the start of the study.

•    Unstable/unmanaged psychotic symptoms requiring active assessment or treatment including medication dose titration (i.e., dose adjustment in the previous 4 weeks or with potential further dose adjustment planned for the following 4 weeks).

*Informed consent*: Site staff will screen potential participants for inclusion into the trial. All participants who match all inclusion criteria and no exclusion criteria will be approached to discuss the trial and provide with the study information sheet. Participants will be given at least 24 hours to consider the trial and informed consent would be taken by an art therapist involved in the trial. As part of the consent procedure participants will be asked if they are happy for intervention sessions to be audio recorded and for their study data to be stored and shared to support future research projects. Participants could decline both of these options and still participate in the trial. Data from participants who have provided consent for their data to be shared for future use will be uploaded to trial record on Open Science Framework data repository.

## Trial intervention

### Interpersonal art psychotherapy

Interpersonal art psychotherapy is a manualised intervention delivered by a trained Health and Care Professions Council (HCPC) registered art psychotherapist. Interpersonal art psychotherapy approach/principles, specific instructions, detailed techniques and intervention delivery approach are described in a standardised therapist manual allowing replication. The intervention is delivered by an art psychotherapist who has completed interpersonal art psychotherapy manual training, treatment fidelity checks, and is receiving clinical supervision ( Hackett *et al.*, 2020). The therapy is scheduled for 12 1-hour individual sessions and 3 optional 1-hour sessions can be added at any time point for personalised support and reasonable adjustments required for a participant with specific communication/learning and/or therapeutic need. Interpersonal art psychotherapy includes seven components arranged as follows; (1) personal goals; (2) coping responses and self-management; (3) relationships; (4) life events; (5) interpersonal themes; (6) imagined future; and (7) final review. Participants have an option to write a letter directly to members of their care team to inform them about the things they are trying to achieve during the intervention and to highlight any personal support needs. Participants completing 5 out of the 7 therapy components listed above will be considered as having completed the intervention.

### Comparator

Within inpatient secure care UC involves assessment and treatment by specialist professionals. The MDT (multi-disciplinary team) model uses the Care Programme Approach (CPA) ( NHS England, 2018a) to coordinate and plan care. MDTs comprise psychiatrists, clinical and forensic psychologists, mental health and ID nursing staff, and Allied Health Professionals (AHPs). MDTs conduct risk assessment/formulation and management, recovery-focused care and/or positive behaviour support (PBS) ( NHS England, 2018a). Patients have access to psychotherapy/psycho-educational work and/or specific offence-related treatment and/or pharmacotherapy treatment. We will identify specific characteristics of UC at the study sites using a standardised pro-forma checklist and will use the TiDieR checklist to describe and present this information ( Hoffmann *et al.*, 2014). This will inform the cost-effectiveness analysis and identify any cross-site variation.

### Compliance and treatment fidelity

Assessing treatment fidelity is important for multi-site studies to ensure that treatments are operationalised and monitored for differentiation, competency and adherence ( Borrelli, 2011). Therapist adherence to the interpersonal art psychotherapy manual within a feasibility study were estimated at 82.25% ( Hackett *et al.*, 2020). All sessions will be audio recorded and a sample of 3 timepoints (sessions 2,7,12) for 3 participants across 9 therapists (27% of sessions) will be rated by raters independent to treatment and any other trial procedures, using the interpersonal art psychotherapy therapist checklist, incorporating tested methods for assessing treatment fidelity ( Hackett *et al.*, 2020).

### Prohibited treatments

Once in the trial, participants are not allowed to take part in any other individual art therapy programmes. Participants who have been randomised to UC only can access the interpersonal art therapy intervention once they have completed the study. This option was included in response to feedback during the Patient and Public Involvement (PPI) consultation process.

## Outcomes measures

### Primary outcome measure(s)

The primary outcome is the frequency/severity of incidents of aggressive behaviour as measured by the Modified Overt Aggression Scale (MOAS) ( Kay *et al.*, 1988). The MOAS will be completed by healthcare staff at baseline, and then again at 19 weeks and 38 weeks post-randomisation. The primary outcome timepoint is at 19 weeks. The MOAS is an observer-rated measure of frequency and severity of aggression (< 10 or > 10 observations over 7 days), (intra-class correlation coefficient (ICC) of 0.94-0.91) ( Kay *et al.*, 1988). Previous research has used the MOAS scale to assess incidents of aggressive and challenging behaviour among adults with intellectual disabilities ( Cohen *et al.*, 2010). The MOAS was found to be a reliable measure for assessing the effectiveness of psychological interventions in people with ID and has been explored in both research ( Oliver *et al.*, 2007) and clinical settings ( Ratey & Gutheil, 1991)

### Secondary outcomes measure(s)

The secondary outcome measures are as follows:

1. Cost-effectiveness of art psychotherapy will be based on estimating the incremental quality-adjusted life years (QALYs) and costs between trial-arms. QALYs will be based on the EuroQoL EQ-5D-3L (self-reported based on the adapted EQ-5D-3L for learning difficulties, proxy-reported based on the EQ-5D-3L Proxy Version-1) and Recovering Quality of Life 10-item version (ReQoL-10; both self and proxy-reported) for deriving the ReQoL Utility Index (ReQoL-UI) as collected at baseline, 19-week, and 38-weeks post-randomisation. Costs will be based on applying unit costs to resource-use compiled using a bespoke resource use measure (RUM; e.g., collecting data on GP appointments, A&E attendance, medication) informed by the patient’s clinicals records, as completed at 19 weeks (i.e., resource use from baseline to 19-weeks) and 38 weeks (i.e., resource use from 20-weeks to 38-weeks) post-baseline.

2. Psychotherapy processes will be evaluated through observer-analysis of transcribed audio-recorded therapy sessions using the Working Alliance Inventory-Observer Ratings Scale (WAI-O), Dual-role Relationship Inventory-Revised (DRI-R), the ‘interpersonal art psychotherapy therapist checklist’, as well as linguistic analysis of change in patients’ use of anger-related words and relational words/pronouns, using the Linguistic Inquiry and Word Count (LIWC) software.

3. Longitudinal changes in aggressive behaviour will be assessed weekly between week 19 and week 38 post-randomisation using the MOAS.

4. Patient distress attributed to psychiatric symptoms as measured by the Brief Symptom Inventory (BSI) Positive Symptom Distress Index (PSDI) ( Derogatis, 1993).

### Participant timeline

A schedule and timeline of procedures for enrolments, intervention, and assessments for participants is shown in Table 1.

**Table 1.** Schedule and timeline of enrolments, interventions and assessment for participants.

Procedures	Screening	Baseline	Randomisation	Treatment Phase	19-week	Weekly assessment	38 week	Ad-Hoc
**Informed consent**	X							
**Eligibility assessment**	X							
**LDSQ**	X							
**Treatment allocation**			X					
**Demographics**		X						
**Medical history**		X						
**Q1 HONOS (WAA or LD)**	X							
**Randomisation**		X						
**Delivery of intervention**				X				
**Compliance**				X				
**MOAS**		X			X	X	X	
**Adapted EQ5D-3L-Self Report**		X			X		X	
**EQD5-3L Proxy**		X			X		X	
**Resource use**					X		X	
**ReQoL-Self Report**		X			X		X	
**ReQoL-Proxy**		X			X		X	
**BSI**		X			X		X	
**Adverse event assessments**								X
**Physician’s Withdrawal Checklist**								X

## Sample size

Our feasibility study ( Hackett *et al.*, 2020) showed a difference of 5 points on the MOAS scale at follow-up with a common standard deviation of 10 points and a correlation of (0.25) between the baseline and post-treatment MOAS scores. We assumed a minimum of 2 therapists per site and 1% intraclass correlation (ICC) of the MOAS scores within the same therapist, we aim to recruit 75 participants in each arm to detect the above difference of 5 points of the MOAS score at follow-up between the two groups with a two-sided significance level of 5% and a power of 80% and an attrition rate of 20%. This calculation is based on the repeated measures ANCOVA.

## Recruitment and withdrawal

A total target of 150 (75 per arm) participants will be recruited. Participants will receive a total of £50 in ‘recognition payment’ during their participation in the trial ( National Institute for Health and Care Research, 2024). The MOAS represents the minimum dataset and where possible trial activities were modular allowing participants to withdraw from individual elements while staying in the trial for other elements. Participants have the right to withdraw consent for participation in any aspect of the trial at any time. Withdrawal rates will be reviewed at monthly Trial Management Group Meetings. If a site has a significantly higher withdrawal rate compared to other sites, the trial team will meet to discuss how to manage requests for withdrawal in way that maintains scientific integrity as well as research ethics. 

## Randomisation

We will randomly allocate participants to interpersonal art psychotherapy or UC in a 1:1 ratio (1 participant allocated to UC for every 1 allocated to the intervention arm) using randomly permuted blocks stratified by sex and diagnosis of psychosis. The final randomisation list will be generated by an independent statistician who will not be involved in the trial and securely uploaded to the randomisation module on the REDCap database. Site staff can register and randomise participants through the RedCap trial database which will be available 24 hours a day.

## Blinding

The trial statistician who will conduct the final data analysis and the research support staff completing the primary outcome assessment will be blind to allocation. Trial statistician will remain blind to treatment allocation during data cleaning and testing of analysis syntax (using dummy randomisation data). Once cleaning has been completed and the analysis syntax has been finalised, treatment allocation will be requested. As the trial art therapist, and Principal Investigator (PI) at site will not be blinded to treatment allocation, there is no planned unblinded or emergency unblinding procedures in place. Any accidental unblinding, i.e. research support staff become unblinded, will be tracked through the trial unblinding log.

## Data collection, management, and analysis

### Data collection

**Figure 1.**
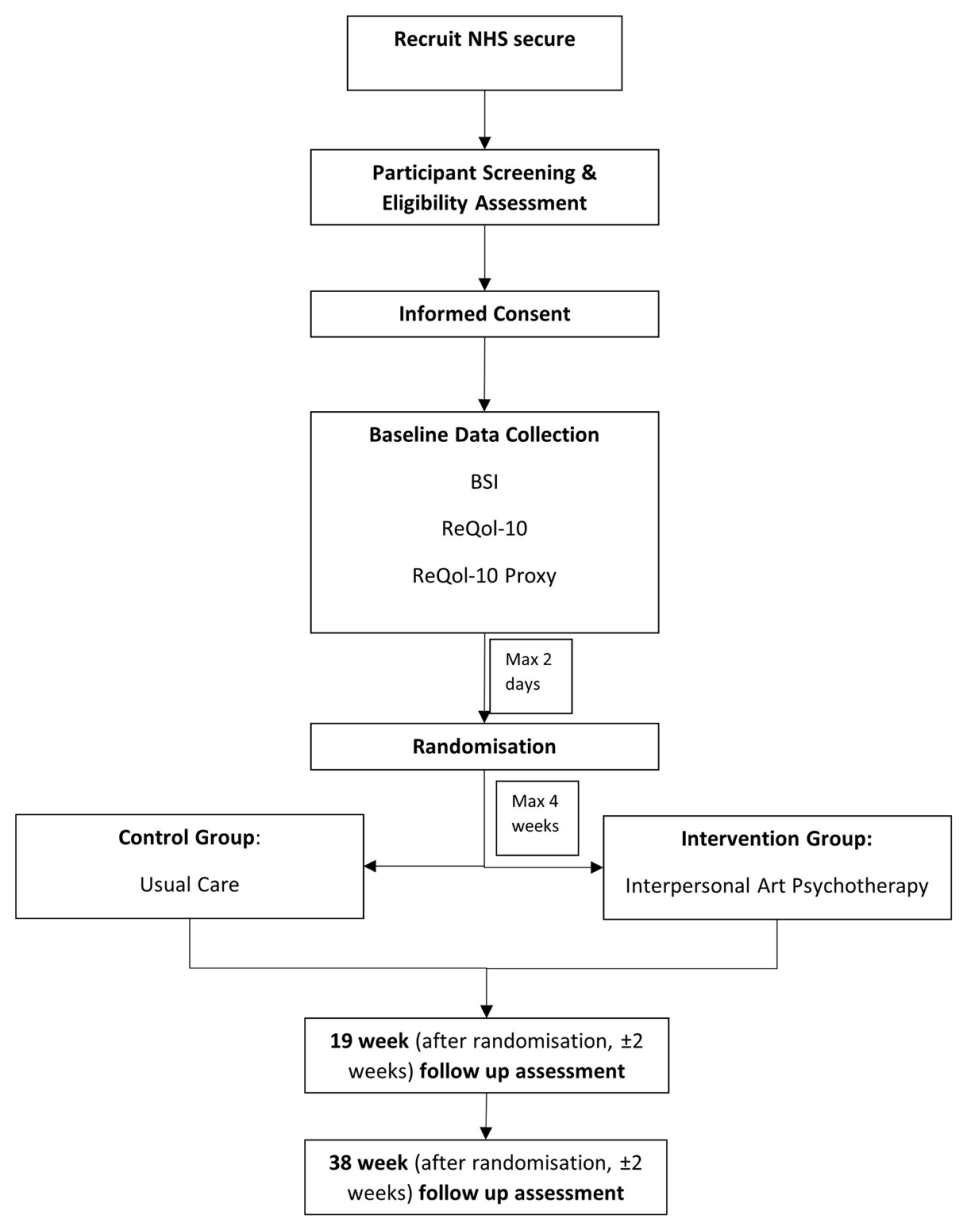
Participant treatment and data collection.

Information regarding participant treatment and data collection is displayed in Figure 1. Trial data will be collected using both electronic data capture (EDC) through REDCap and paper forms. Participant facing questionnaires (quality of life questionnaires, and BSI) will be completed on paper and then entered into the EDC system. The system will include all the necessary validation to ensure a level of data quality. All data management processes, including quality control checks of paper forms entered onto the database, will be fully detailed in the trial data management plan.

### Data management

***Missing, unused & spurious data***. Missing data will be investigated for cause and extent. If required, the Statistical and Health Economic Analysis Plan (SHEAP) will detail the methods to be used to deal with missing data. Any missing data will be queried with the sites using the resolution workflow in REDCap to ensure that this is missing, and a code will be assigned using the database.

***Procedures for reporting deviation(s) from the original SHEAP***. These will be submitted as substantial amendments where applicable and recorded in subsequent versions of the protocol and SHEAP.

***Inclusion in analysis***. The primary analysis for the trial will be performed on an intention-to-treat basis therefore all participants who are randomised will be included in the analysis and so this will be conducted under the treatment policy estimate strategy.

As the per the Good Clinical Practice (GCP), the definition of Source Data is *“All information in original records and certified copies of original records of clinical findings, observations or other activities in a clinical trial necessary for the reconstruction and evaluation of the trial. Source data are contained in source documents.”* For any data element, there can only be one set of source data , and this will be defined in the site source data agreement.

The source data information for SCHEMA will come from a variety of sources (see Table 2). Data will be collected using an electronic Case Report Form system (CRF) with paper CRF back up. There will also be data collected from participants’ medical notes and patient-reported questionnaires. All delegated staff at the sites will receive appropriate training to complete the CRFs.

**Table 2.** Trial and source data information.

Trial data	Source Data			
	Participant medical notes	Electronic System	Questionnaire	SAE form
Medical History	X			
Concurrent Medications	X			
Adverse events	X			X
MOAS			X	
EQ5D-3L: Self			X	
EQ5D: Proxy		X		
Use of Service		X		
ReQOL: Self			X	
ReQOL: Proxy		X		
BSI			X	

***Protocol/GCP non-compliance***. The PI will report any divergencies from the trial protocol or principles of GCP to the central trial team by email as soon as they become aware of it. The central trial team will review the divergence against the protocol, and local standard operating procedures (SOP) to determine the nature and severity. All divergences will be reported to the Sponsor.

***Sponsor details***. Cumbria, Northumberland, Tyne and Wear NHS Foundation Trust, St. Nicholas Hospital Jubilee Road Gosforth Newcastle upon Tyne NE3 3XT.Contact email: CNTWSponsorManagement@cntw.nhs.uk

The sponsor did/will not have any involvement in study design, data collection, management, analysis and interpretation of data, or writing of the report.

## Statistical methods

### Main analysis

***Outcome/effectiveness analysis***. The analysis of the primary outcome MOAS will be performed using analysis of covariance, modelling 19 weeks follow-up MOAS score controlling for baseline MOAS score. Reflecting the sample size calculation analyses will be undertaken with 2-level hierarchical models with participants clustered within therapists. Secondary outcomes will be analysed in a similar way. Multiple imputations will be used in case of missing values in scores. The results will be summarised using point estimates, 2-sided 95% confidence intervals and p-values. Data analysis will be in accordance with a pre-specified statistical analysis plan.

***Psychotherapy process analysis***. It has been recommended that future research on psychological interventions for institutional aggression should include an assessment of patient characteristics and interpersonal styles that facilitate participation and progress during treatment ( Daffern *et al.*, 2008). These process analyses will be conducted on samples of treatment sessions for each patient, to reflect the segments of the treatment and change over time. The reading of the session transcripts together with the process coding of the WAI-O and DRI-I are expected to take about 1.5 hours per session.

***Working Alliance Inventory-Observer version (WAI-O)***. The WAI-O ( Darchuk *et al.*, 2000) is a 12-item measure of the working alliance as measured by an observer. This brief WAI-O was developed from the original 36 item Working Alliance Inventory (WAI) ( Horvath & Greenberg, 1989). The WAI-O contains three subscales: agreement on tasks, agreement on goals, and development of bonds, each with 4 items rated on 7-point Likert-type scales (7 = high). The client and therapist reported WAI was adapted to fit an observer perspective (the WAI-O) by altering the pronouns ( Tichenor & Hill, 1989). The WAI-O will be rated by two independent judges for the sessions. The working alliance score used in analyses will be averaged between the two raters across a sample of sessions for each patient.

***Dual Role Relationship Inventory (DRI-R-SF)***. The Dual-Relationship Inventory-Short Form is a 9-item abbreviated versions of the Dual Role-Relationship Inventory (30 items) that assesses the quality of relationships in mandated treatment settings ( Gochyyev & Skeem, 2019). The DRI-SF is a nine-item relationship instrument with items rated on a 7-point Likert-type-scale: 1 = never, 2 = rarely, 3 = occasionally, 4 = sometimes, 5 = often, 6 = very often, and 7 = always. A higher DRI-SF total score indicates a better quality of client-supervising officer relationship. The DRI-R-SF assesses three relationship aspects; caring-fairness, toughness, and trust. The DRI-R-SF has shown high internal consistency and predictive validity for probation violations and revocation, and moderate inter-item correlations.

***Linguistic Analysis of Word Count (LIWC-2022)***. We will apply the text analysis software LIWC-2022 to the transcripts of the sessions ( LIWC-22, 2022). We specifically will analyse the change in frequency of patients’ anger-related word use over the course of treatment, and the change in affiliation/pronoun use. As with the other two process measures, we will use the transcripts from a sample of sessions per treatment as a proxy of the treatment process as a whole.

### Sub-group & interim analysis

A sub-group analysis of differences between therapy responders and non-responders will be completed. Full details of the analysis will be included in the primary trial SHEAP.

### Qualitative analysis

The qualitative analysis will include a minimum of 20 audio-recorded and transcribed interviews integrated into the RCT ( Davis *et al.*, 2019). A five-step framework analysis will be completed, including data familiarisation, identifying a framework, indexing, charting, and mapping and interpretation ( Ritchie & Spencer, 1994) from a minimum sample of *n*=20 qualitative interviews. Based upon guidance on the development of complex interventions to improve health, qualitative interviews and analysis will contribute to a process evaluation of study retention, experiences of the intervention, fidelity, dose, and reach ( O'Cathain *et al*., 2019). We will invite a minimum of to 10 participants (recruited from both arms) and 10 study therapists to engage in semi-structured qualitative interviews. A minimum of 10 interview participants can be considered sufficient where the aim of the research is to reach an in-depth understanding of participant experience, rather than to seek generalizability of findings. Data saturation can often be achieved with a sample size of 6 to 12 participants ( Guest *et al.*, 2006), and furthermore, evidence suggesting that insightful and meaningful analysis can be achieved from relatively small sample sizes within health research where samples have been purposively selected ( Pons-Vigués *et al*., 2019). There will be no cut-off for sample size of participants included for interview, all study participants and therapists who express an interest in taking part in an interview will be given the opportunity to participate. Full details of analysis will be included in the qualitative analysis plan.

Interview schedules for study therapists will focus on their experiences of intervention delivery, their experiences of training and supervision, seeking examples from therapists about their ‘in-therapy’ responses from the participants, and implications for clinical practice implementation. Participant interview schedules will focus on the participants experience of taking part in research, their experiences and reflections on art making, therapy and their relationship with the therapist within sessions.

### Cost-effectiveness analysis

A within-trial cost-effectiveness analysis will be conducted using the same statistical methods as the primary outcome (i.e., 2-level hierarchical model) ( Glick *et al.*, 2014). Costs will focus on the incremental costs/resource-use of providing art psychotherapy relative to UC and downstream resource-use collected via a bespoke resource-use measure (RUM) proforma based on patients’ care records (e.g. wider staff engagement and medications). Completion of the RUM involves research staff checking the patient notes for what resources the patient has used based on the proforma, which they then confirm with ward staff. Costs will be estimated based on attaching unit costs to resource-use data, with unit costs sourced from commonly used UK sources ( BMJ, 2024; Jones *et al.*, 2024; NHS England, 2023). Incremental effectiveness will be based on two preference-based measures: EQ-5D-3L ( EuroQol, 2024), and ReQoL-UI derived from the ReQoL-10 ( Keetharuth *et al.*, 2024). These measures will include both a self-reported (i.e., by the patient) and proxy-completed (i.e., by ward staff) version. For proxy-completion, we have specified that at each time point both the EQ-5D-3L and ReQoL-10 should be completed by the same person (i.e., same ward staff member) and where possible it should be the same person across time points, but we recognise that might not always be possible. We will record who has completed the proxy-response. For the EQ-5D-3L, the self-reported version is based on the adapted EQ-5D-3L for adults with mild to moderate learning disabilities ( O’Dwyer *et al.*, 2024); whereas the proxy-version is the EQ-5D-3L Proxy Version-1 ( EuroQol, 2024). There are no adapted or proxy versions of the ReQoL-10; therefore, the self-reported and proxy-reported versions are both the traditional ReQoL-10. The EQ-5D-3L and ReQoL-UI both have a utility index (UI) which can be used to elicit quality-adjusted life years (QALYs). The UI for the EQ-5D-3L and ReQoL-UI will be based on the relevant UK value set, respectively ( Dolan, 1997; Keetharuth *et al.*, 2021).

These measures have underlying conceptual and methodological considerations relevant to the patient population and intended outcomes from art psychotherapy improvements. As such, the choice of preference-based measure as the ‘primary’ outcome for the economic evaluation will be based on a within-study psychometric analysis. We are unaware of any previous psychometric results that aid with understanding the appropriateness of the EQ-5D-3L and ReQoL measures in our trial’s patient population of interest; thus, our proposed psychometric analyses can aid such measure justification in our trial and future trials in a similar patient population where similar outcomes are of interest. This post-hoc psychometric analysis will be conducted based on construct validity (i.e., correlation and effect sizes relative to clinical outcomes) and responsiveness (i.e., standardised response means, and floor and ceiling effects) relative to the other primary and secondary outcome measures, as has been done to judge the relative appropriate use of the EQ-5D (-5L) and ReQoL-UI for cost-effectiveness analyses previously ( Franklin *et al.*, 2021; Franklin *et al.*, 2022). The cost-effectiveness results using all measures will be reported and discussed; the psychometric analyses will be conducted and the choice of primary measure will be detailed in our SHEAP before conducting the cost-effectiveness analyses.

Point-estimate cost-effectiveness will be presented using incremental cost-effectiveness ratios. Bootstrapping will be used to report bootstrapped standard errors, and to estimate the probability of cost-effectiveness relative to a range of cost-effectiveness thresholds (including NICE’s £20,000 to £30,000 per QALY threshold) to be presented using cost-effectiveness planes and cost-effectiveness acceptability curves ( Glick *et al.*, 2014). Sensitivity analyses will be used to assess point estimate uncertainty.

## Monitoring

### Trial management

***TMG (Trial Management Group) and Advisory Group (AG)***. The TMG will have regular meetings (every 4–6 weeks) prior to and during the study (Y1–4) chaired by the Chief Investigator (CI) with CTR support. TMG members will sign up to the remit and conditions set out in the TMG Charter.

***TSC (Trial Steering Committee)***. This is an low-risk trial therefore a Independent Data Monitoring Committee (IDMC) will not be convened, unless recommended by the Trial Steering Committee (TSC). TSC membership will comprise of members who are independent of the trial taking up positions as chair, statistician, expert therapist, and a public member having signed-up to the remit and conditions as set out in the TSC Charter.

### Quality control and assurance

***Monitoring***. Trial specific monitoring activity is determined by the risks outlined within the clinical trial risk assessment and the overall determination of risk. All activities will be fully documented within the trial monitoring plan and deviations appropriately recorded by the trial and sponsorship team.

### Harms

***Safety reporting***. It is expected that all Serious Adverse Events (SAEs) be reported no later than 24 hours after knowledge of the event by the site PI or delegated site staff, unless an exemption has been specified within the protocol. Safety reporting terms for the trial are shown in Table 3 and guidance for determining intervention causality is shown in Table 4.

**Table 3.** Definitions of terms relating to safety reporting.

Term	Definition
**Adverse Event (AE)**	Any untoward medical occurrence that impacts the intervention, day to day activities, or requires medical intervention in a participant or clinical trial participant administered an intervention which is not necessarily caused by or related to that product
**Serious Adverse Event** **(SAE)**	Any adverse event that - Results in death Is life-threatening * Required hospitalisation or prolongation of existing hospitalisation ** Results in persistent or significant disability or incapacity Consists of a congenital anomaly or birth defect Other medically important condition***
**Serious Adverse Reactions** **(SARs)**	Any SAE occurring in a clinical trial participant for which there is a reasonable possibility that it is related to the intervention.
**Suspected Unexpected** **Serious Adverse Reactions** **(SUSARs)**	A SAR, the nature and severity of which is not consistent with the Reference Safety Information (RSI) for the intervention.

*Note: The term ‘life-threatening’ in the definition of serious refers to an event in which the trial participant was a risk of death at the time of the event, or it is suspected that used of continued use of the product would result in the subjects death; it does not refer to and event which hypothetically might have caused death if it were more severe. **Note: Hospitalisation is defined as an inpatient admission, regardless of the length of stay even if the hospitalisation is a precautionary measure for continued observation. Pre-planned hospitalisation e.g. for pre-existing conditions which have not worsened, or elective procedures, does not constitute and SAE.

**Table 4.** Guidance for determining intervention causality.

Relationship	Description	Reasonable possibility that the SAE may have been caused by the intervention?
**Unrelated**	There is no evidence of any casual relationship with the intervention.	No
**Unlikely**	There is little evidence to suggest there is a causal relationship with the intervention (e.g. the event did not occur within a reasonable time after administration of the trial medication). There is another reasonable explanation of the event (e.g. the participant’s clinical condition, other concomitant treatment).	No
**Possible**	There is some evidence to suggest a causal relationship with the intervention (e.g. because the event occurs within a reasonable time after administration of the trial medication). However, the influence of other factors may have contributed to the event (e.g. the participant’s clinical condition, other concomitant treatments).	Yes
**Probable**	There is evidence to suggest a causal relationship and the influence of other factors is unlikely.	Yes
**Definite**	There is clear evidence to suggest a causal relationship and other possible contributing factors can be ruled out.	Yes

***Trial Specific SAE Reporting requirements***. There is an additional event that is being classed as an SAE for the purposes of this trial and therefore must follow the SAE reporting guidelines:

- Incidences of self-harm recorded in clinical/case notes.
- The local site PI or appropriately delegated individual will be responsible for assessing the causality of the event, i.e. whether the event was caused by the intervention or another significant factor.
- All SAE will be reviewed for expectedness by CI or another appropriately delegated individual who is independent of the site.

### Auditing

The trial is participant to inspection by Research Ethics Committee (REC) as the regulatory body. The trial may also be participant to inspection and audit by Cumbria, Northumberland, Tyne, and Wear NHS Foundation Trust, under their remit as Sponsor.

### Ethics and dissemination

This protocol and related trial documents were reviewed by the London-City & East REC and received full approval on 13/01/2023 (REC ID: 23/LO/0026; IRAS project ID: 319325). All participants will be approached to provide written consent prior to being entered into the trial.

### Confidentiality

The CTR will act to preserve participant confidentiality and will not disclose or reproduce any information by which participants could be identified, except where specific consent is obtained. Data will be stored securely and will be registered in accordance with the General Data Protection Regulation 2016 and Data Protection Act 2018. The data custodian is Cumbria, Northumberland, Tyne and Wear NHS Foundation Trust. Select trial data will be available on the Open Science Framework data repository.

### Dissemination policy

***Publication policy***. All publications and presentations relating to the trial will be detailed in the publication policy which will be drafted and authorised by the TMG. It will state principles for publication, describe a process for developing output, contain a map of intended outputs and specify a timeline for delivery. The publication policy will respect the rights of all contributors to be adequately represented in outputs (e.g. authorship and acknowledgements) and for the trial to be appropriately acknowledged. Authorship of parallel studies initiated outside of the TMG will be according to the individuals involved in the project but must acknowledge the contribution of the TMG and the CTR. We will include PPI consultees in the development of accessible ‘easy read’ summaries of results and accessible materials.

## Data Availability

No underlying data are associated with this article. The Statistical Analysis Plan (SAP) will be made publicly available in an open-source publication prior to data analysis being started. The CTR will act to preserve participant confidentiality and will not disclose or reproduce any information by which participants could be identified, except where specific consent is obtained. Data will be stored securely and will be registered in accordance with the GDPR 2016 and DPA 2018. The data custodian is Cumbria, Northumberland, Tyne and Wear NHS Foundation Trust. Select trial data will be available on the Open Science Framework data repository. ISRCTN registry: Secure care hospital evaluation of art therapy ( ISRCTN registry, 2024) https://doi.org/10.1186/ISRCTN57406593 This project contains the following extended data: Participant information sheets Protocol file OSF: SPIRIT Checklist ( Chan *et al.*, 2013) For ‘Secure care hospital evaluation of manualised interpersonal art-psychotherapy (SCHEMA): A randomised controlled trial protocol.’ https://doi.org/10.17605/OSF.IO/V53F4 ( Foscarini-Craggs, 2025) Data are available under the terms of the Creative Commons Zero "No rights reserved" data waiver (CC0 1.0 Public domain dedication).
