# Supplementary material for: Secure care (forensic) hospital evaluation of manualised interpersonal art-psychotherapy (SCHEMA): A randomised controlled trial protocol
Source: NIHR Open Res. 2025 May 28;5:21. Originally published 2025 Mar 7. [Version 2] doi: 10.3310/nihropenres.13801.2 (PMC12000804; doi:10.3310/nihropenres.13801.2)
Supplement: Peer review history

# Reviewer report: Reviewer response for version 1

**DOI:** 10.3310/nihropenres.14993.r34953

**Published:** 2025-04-15

**Authors:** Emma Gentle

**Affiliations:** 1 Centre of Disability Studies, The University of Sydney School of Medicine, Sydney, New South Wales, Australia; 2 Art Therapy, Western Sydney University School of Social Sciences, Parramatta South, New South Wales, Australia; 3 Health, Southern Cross University, New South Wales, Australia

Review

*Secure care hospital evaluation of manualised interpersonal art-psychotherapy (SCHEMA): A randomised controlled trial protocol *is methodologically complex yet clear enough to be repeatable.

 The abstract described the study. It uses fairly accessible language to describe the study. I would reword this sentence: *A feasibility randomised controlled trial of interpersonal art psychotherapy showed that assessment of key feasibility objectives were met and the trial procedures were acceptable, indicating progression to a definitive trial.*  

 E.g. A feasibility study was conducted of a controlled trial of utilising interpersonal art psychotherapy and showed that assessment of the key feasibility objectives were met and the trial procedures were acceptable, indicating progression to a definitive trial. This is an important factor when designing an intricate and sophisticated research project with people who do not work in the scientific field, which often applies to the participants.

 The introduction is clearly and succinctly outlined with relevant research incorporated. This section segued easily into the more involved yet comprehensive methods section.     

 The method questions helped this section by giving it clarity and order, making it easy to follow. The inclusive research design with a randomised control study method strengthens the research. The inclusivity of the research is  described in enough detail to show the authenticity of the authors in ensuring ethical protocols are not just followed but expanded upon. This helps show other researchers the value in inclusive research.

 Title: For someone outside the UK it may be useful to put forensic in brackets after secure care

 in paragraph 2 in the  Introduction, I suggest you to please revise the sentences - 

***Background: and/or learning disability and in paragraph 2 in the  Introduction- unless this is a way of writing and/or that I am unfamiliar with".***

***Background: a personality disorder, and/a learning disability*** *- change to and/or*

***Introduction: a personality disorder, and/a learning/intellectual disability (ID)*** *- change to and/or*

 Introduction

 Third paragraph: considered long stayers

 Methods

 How were people involved?

 Prohibited treatments section does not flow easily

 Statistical Methods are not my speciality. However, this section is described well.

 The qualitative analysis section could be clearer in its language and/or have a diagram that clarifies the process

 P12 **Note: Hospitalisation is defined as an impatient admission, regardless of the length of stay even if the hospitalisation is a precautionary measure for continued observation. Pre-planned hospitalisation e.g. for pre-existing conditions which have not worsened, or elective procedures, does not constitute and SAE.

 I found this paper to be thorough, carefully conceived and with demonstrable inclusion of participants. I am looking forward to reading the paper on completion of the project. It sounds valuable to the participants and to the incarceration systems for people within secure hospital settings globally.

Is the study design appropriate for the research question?

Yes

Is the rationale for, and objectives of, the study clearly described?

Yes

Are sufficient details of the methods provided to allow replication by others?

Yes

Are the datasets clearly presented in a useable and accessible format?

Not applicable

Reviewer Expertise:

Art therapy, community arts therapy, qualitative and inclusive research, mental health, neurodiversity

I confirm that I have read this submission and believe that I have an appropriate level of expertise to confirm that it is of an acceptable scientific standard.

# Response

**Published:** 2025-05-09

**Authors:** Simon Hackett

**Affiliations:** Population Health Sciences, Newcastle University, Newcastle upon Tyne, England, UK

Title: For someone outside the UK it may be useful to put forensic in brackets after secure care

**Response: **Additional text, (forensic), has been added to the title.

 in paragraph 2 in the Introduction, I suggest you to please revise the sentences - 

*Background: and/or learning disability and in paragraph 2 in the Introduction- unless this is a way of writing and/or that I am unfamiliar with".*

*Background: a personality disorder, and/a learning disability - change to and/or*

*Introduction: a personality disorder, and/a learning/intellectual disability (ID) - change to and/or*

**Response:** Thank you, wording has been changed as suggested.

 Introduction

 Third paragraph: considered long stayers

**Response:** Thank you, wording has been changed as suggested.

 Methods

 Prohibited treatments section does not flow easily

**Response: **Thank you, this section has been edited to improve readability.

 The qualitative analysis section could be clearer in its language and/or have a diagram that clarifies the process

**Response: **The language in the qualitative analysis section has been revised and edited.

 P12 **Note: Hospitalisation is defined as an impatient admission.

**Response:** Thank you, wording has been changed as suggested.

# Reviewer report: Reviewer response for version 1

**DOI:** 10.3310/nihropenres.14993.r34956

**Published:** 2025-04-10

**Authors:** Dominik Havsteen-Franklin

**Affiliations:** 1 Brunel University London, London, UK

This protocol is methodologically robust, ethically grounded, and addresses a significant clinical gap in forensic care for individuals with mild intellectual disability and borderline intellectual functioning. However a few areas could benefit from refinement:

**Comparator (UC)**

 The comparator is defined as multidisciplinary team-based usual care (UC), yet significant variability is likely across NHS and independent sector sites. This could dilute treatment effects or complicate interpretation unless carefully accounted for in the analysis ^1^(Ref-1). It also would be helpful to know whether site-level variability will be modelled ^2,3^.

**Attrition Management**

 Given the extended follow-up period (up to 9 months), more detail on how attrition will be monitored and managed would be helpful, particularly regarding safeguarding statistical power and data integrity ^4^.

**Secondary Outcome Measures**

 While ReQoL UI and EQ-5D-3L are appropriate and widely used, their responsiveness and conceptual alignment with this specific population could be further justified ^5^. Further, in the event of discordant results, it would be useful to clarify how these would be interpreted in the analysis.

**Data Governance and Accessibility**

 A minor point, but explicitly stating where the data will be deposited and how this aligns with ethical and governance approvals for NIHR open access would be beneficial.

**Workforce and Implementation Considerations**

 It might be helpful to describe how differences in therapist practices and contextual implementation factors might influence outcomes or be considered in future workforce planning - especially in consideration of scalability ^6,7,8,9^.

**Qualitative Component**

 The qualitative sample size (n=20 across participants and therapists) may be sufficient for thematic saturation in focused studies. However, given the complexity of the intervention and setting, further justification could be given as to why this number is considered sufficient for addressing the intended implementation and experiential questions ^10,11,12,13^.

**Conclusion**

 This protocol responds to a critical evidence gap using a thoughtful and well-articulated trial design. The recommendations above are intended to enhance clarity and utility of the protocol but are not prerequisites for publication. The protocol design aligns well with the standards expected for NIHR Open Research and is ready for indexing in its current form.

Is the study design appropriate for the research question?

Yes

Is the rationale for, and objectives of, the study clearly described?

Yes

Are sufficient details of the methods provided to allow replication by others?

Yes

Are the datasets clearly presented in a useable and accessible format?

Not applicable

Reviewer Expertise:

Arts Psychotherapies; NHS Clinical Practice; Complex Interventions in Mental Health; Practice-Based Research; Psychotherapy Methodology; Arts and Health.

I confirm that I have read this submission and believe that I have an appropriate level of expertise to confirm that it is of an acceptable scientific standard.

## References

[1] : Site-level variability in the processes of care and outcomes over time among patients with COVID-19 and myocardial injury: Insights from the American Heart Association's COVID-19 Cardiovascular Disease Registry. *Am Heart J Plus* .2023;27: 10.1016/j.ahjo.2023.100265 100265 10.1016/j.ahjo.2023.100265 36779177 PMC9894823

[2] : Modeling site effects in the design and analysis of multi-site trials. *Am J Drug Alcohol Abuse* .2011;37(5) : 10.3109/00952990.2011.600386 383-91 10.3109/00952990.2011.600386 21854281 PMC3281513

[3] : Identifying research priorities for effective retention strategies in clinical trials. *Trials* .2017;18(1) : 10.1186/s13063-017-2132-z 406 10.1186/s13063-017-2132-z 28859674 PMC5580283

[4] : Psychometric assessment of EQ-5D-5L and ReQoL measures in patients with anxiety and depression: construct validity and responsiveness. *Qual Life Res* .2021;30(9) : 10.1007/s11136-021-02833-1 2633-2647 10.1007/s11136-021-02833-1 33835414 PMC8034045

[5] : Disentangling the Therapist Effect: Clustering Therapists by Using Different Treatment Outcomes. *Adm Policy Ment Health* .2024;51(5) : 10.1007/s10488-024-01365-3 769-779 10.1007/s10488-024-01365-3 38512559 PMC11379780

[6] : What makes us the same? What makes us different? Development of a shared model and manual of group therapy practice across art therapy, dance movement therapy and music therapy within community mental health care. *The Arts in Psychotherapy* .2021;72: 10.1016/j.aip.2020.101747 10.1016/j.aip.2020.101747

[7] : Growth curves of common factors in psychotherapy: Multilevel growth modelling and outcome analysis. *Clin Psychol Psychother* .2023;30(5) : 10.1002/cpp.2864 1095-1110 10.1002/cpp.2864 37204078

[8] : Ensuring treatment fidelity in intervention studies: Developing a checklist and scoring system within a behaviour change paradigm. *Int J Lang Commun Disord* .2024;59(1) : 10.1111/1460-6984.12955 379-395 10.1111/1460-6984.12955 37715525 PMC13504697

[9] : Guidance on how to develop complex interventions to improve health and healthcare. *BMJ Open* .2019;9(8) : 10.1136/bmjopen-2019-029954 e029954 10.1136/bmjopen-2019-029954 31420394 PMC6701588

[10] : Qualitative evaluation of a complex intervention to implement health promotion activities according to healthcare attendees and health professionals: EIRA study (phase II). *BMJ Open* .2019;9(3) : 10.1136/bmjopen-2018-023872 e023872 10.1136/bmjopen-2018-023872 30904842 PMC6475230

[11] : Code Saturation Versus Meaning Saturation: How Many Interviews Are Enough?. *Qual Health Res* .2017;27(4) : 10.1177/1049732316665344 591-608 10.1177/1049732316665344 27670770 PMC9359070

[12] : Characterising and justifying sample size sufficiency in interview-based studies: systematic analysis of qualitative health research over a 15-year period. *BMC Med Res Methodol* .2018;18(1) : 10.1186/s12874-018-0594-7 148 10.1186/s12874-018-0594-7 30463515 PMC6249736

[13] : Partially nested designs in psychotherapy trials: A review of modeling developments. *Psychother Res* .2017;27(4) : 10.1080/10503307.2015.1114688 425-436 10.1080/10503307.2015.1114688 26686878

# Response

**Published:** 2025-05-09

**Authors:** Simon Hackett

**Affiliations:** Population Health Sciences, Newcastle University, Newcastle upon Tyne, England, UK

**Comparator (UC)**

 The comparator is defined as multidisciplinary team-based usual care (UC), yet significant variability is likely across NHS and independent sector sites. This could dilute treatment effects or complicate interpretation unless carefully accounted for in the analysis ^1^(Ref-1). It also would be helpful to know whether site-level variability will be modelled ^2,3^.

**Response:** Site-level variability will not be modelled. To describe potential site variability, as set out under the protocol heading ‘Comparator’, *‘* *We will identify specific characteristics of UC at the study sites using a standardised pro-forma checklist and will use the TiDieR checklist to describe and present this information ( Hoffmann et al., 2014).’*

**Attrition Management**

 Given the extended follow-up period (up to 9 months), more detail on how attrition will be monitored and managed would be helpful, particularly regarding safeguarding statistical power and data integrity ^4^.

**Response:** Attrition rates will be regularly monitored at TMG meetings and if a site has an unusually high withdrawal rate, the trial team will meet with them to discuss. This information has been added to the recruitment and withdrawal section.

**Secondary Outcome Measures**

 While ReQoL UI and EQ-5D-3L are appropriate and widely used, their responsiveness and conceptual alignment with this specific population could be further justified ^5^. Further, in the event of discordant results, it would be useful to clarify how these would be interpreted in the analysis.

**Response:** We are unaware of any existing research that has examined the psychometric properties of the ReQoL-UI and EQ-5D-3L in the specific patient population of interest for our trial. This is part of the rational for the proposed psychometric analyses using our trial data that will be conducted and reported before the economic evaluation is conducted. In the case of discordant results based on the ReQoL-UI and EQ-5D-3L, we will reflect on the psychometric results to explain this discordance. For example, our proposed assessment of construct validity between the MOAS and BSI and ReQoL and EQ-5D-3L will aid with understanding the “conceptual alignment” (as suggested by the reviewer) between these measures (noting the MOAS is our primary outcome, thus a key measure of interest for our psychometric assessment), and our assessment of responsiveness / sensitivity (across and between measures) will aid with understanding “responsiveness” (as suggested by the reviewer). As such, the psychometric analyses will provide the evidence required to address such justifications (or not) for using the ReQoL-UI and EQ-5D-3L in our trial and for future trials.

 We have added the following paragraph to the ‘cost-effectiveness’ section of the paper to address the reviewer’s comment: “We are unaware of any previous psychometric results that aid with understanding the appropriateness of the EQ-5D-3L and ReQoL measures in our trial’s patient population of interest; thus, our proposed psychometric analyses can aid such measure justification in our trial and future trials in a similar patient population where similar outcomes are of interest.”

**Data Governance and Accessibility**

 A minor point, but explicitly stating where the data will be deposited and how this aligns with ethical and governance approvals for NIHR open access would be beneficial.

**Response:** We recognise and support ensuring that data is made available for future research. Participants had the option to consent for their data to shared for future research. We have added a sentence to the informed consent section that explains that data from participants have consent for their data to be shared will be uploaded to the Open Science Framework data repository. A similar sentence has also been added to the data availability section.

**Workforce and Implementation Considerations**

 It might be helpful to describe how differences in therapist practices and contextual implementation factors might influence outcomes or be considered in future workforce planning - especially in consideration of scalability ^6,7,8,9^.

**Response:** This topic, ‘workforce and implementation considerations’ does not form part of the current study protocol. We will report upon ‘Compliance and treatment fidelity’, and therefore describe any differences in manual adherence by therapists during intervention delivery that might influence outcomes.

**Qualitative Component**

 The qualitative sample size (n=20 across participants and therapists) may be sufficient for thematic saturation in focused studies. However, given the complexity of the intervention and setting, further justification could be given as to why this number is considered sufficient for addressing the intended implementation and experiential questions ^10,11,12,13^.

**Response:** Further justification for sample size has been included, with additions made to the qualitative analysis. Additional supporting references - Pons-Vigués M, Berenguera A, Coma-Auli N, March S, et al. (2019) and - Guest, G., Bunce, A., & Johnson, L. (2006) have now been included.
